# Identifying two pathogenic variants in a patient with pigmented paravenous retinochoroidal atrophy

**DOI:** 10.1515/biol-2022-0532

**Published:** 2023-01-16

**Authors:** Zeyuan Liu, He Wang, Xiaoli He, Dan Tao, Li Li

**Affiliations:** Department of Ophthalmology, Kunming Children’s Hospital, No. 288, Qianxing Road, Xishan District, Kunming 650228, China; Department of Ophthalmology, Kunming Children’s Hospital, Yunnan Institute of Pediatrics, Yunnan Province Clinical Research Center for Children’s Health and Disease, Yunnan Key Laboratory of Children’s Major Disease Research, No. 288, Qianxing Road, Xishan District, Kunming 650228, China

**Keywords:** *RPGRIP1*, pigmented paravenous retinochoroidal atrophy, whole-exome sequencing, pathogenic variants

## Abstract

Little is known about the genetic background of pigmented paravenous retinochoroidal atrophy (PPRCA) due to rarity of patients. In this study, we identified two pathogenic variants in *RPGRIP1* in a 2-year-old boy with PPRCA screened by whole-exome sequencing (WES). The patient presented to our department with photophobia for 17 months, and then he underwent fundus photography and fluorescein fundus angiography. Genomic DNA was extracted from peripheral blood of the proband and the parents. Trio-WES strategy was utilized to identify the causal variants from the proband and the parents, followed by validation based on Sanger sequencing. The patient was finally diagnosed with PPRCA after differential diagnosis. Two heterozygous pathogenic variants were detected by WES according to the American college of medical genetics and genomics guidelines, including NM_020366.4: c.2592T > G: p.Y864* and NM_020366.4: c.154C > T: p.R52* in *RPGRIP1* located in exon 17 and exon 3, leading to termination codon, respectively. This is the first study reporting pathogenic variants within *RPGRIP1* as causal for PPRCA.

## 1 Introduction

Pigmented paravenous retinochoroidal atrophy (PPRCA) is a rare disease featured by bilateral retinochoroidal atrophy and pigmentation along the retinal veins [1]. In clinical practice, the diagnosis is mainly relied on the typical fundus manifestations, but it is still a challenge as most patients are usually asymptomatic [2].

To date, the pathogenesis of PPRCA is still not well defined due to rarity of patients. Most PPRCA patients occur sporadically even though there are cases with an apparent family history [3]. Up to now, approximately 100 cases with PPRCA have been reported with considerable variability in the extent and degree in the affected retina [4]. Recently, whole-exome sequencing (WES) has been utilized for screening pathogenic variants of various genetic diseases [5], but few studies have been carried out to investigate the genetic pathogenesis of PPRCA based on WES. In this study, we reported a PPRCA case with two pathogenic variants of *RPGRIP1* screened by WES based on American college of medical genetics and genomics (ACMG) criteria. To our best knowledge, this is the first study reporting pathogenic variants in *RPGRIP1* related to the pathogenesis of PPRCA.

## 2 Case presentation

**Figure 1:**
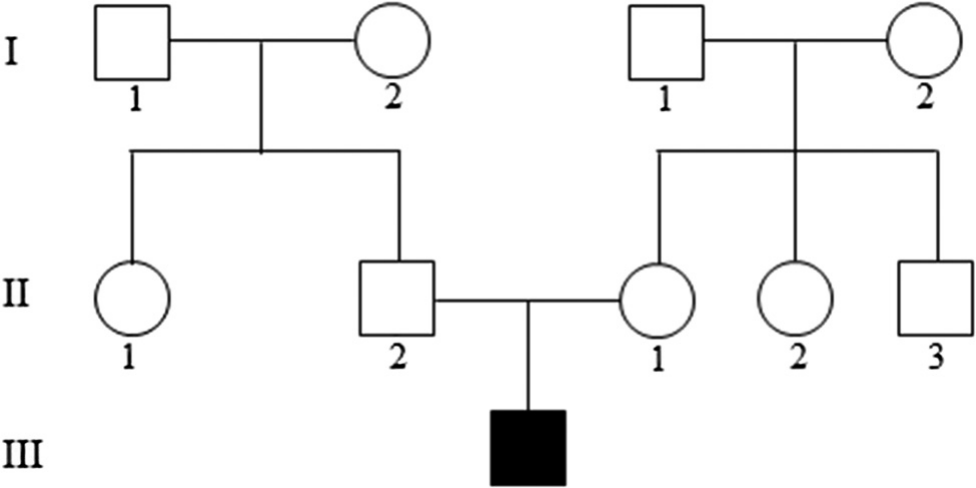
Pedigree of the patient.

**Figure 2:**
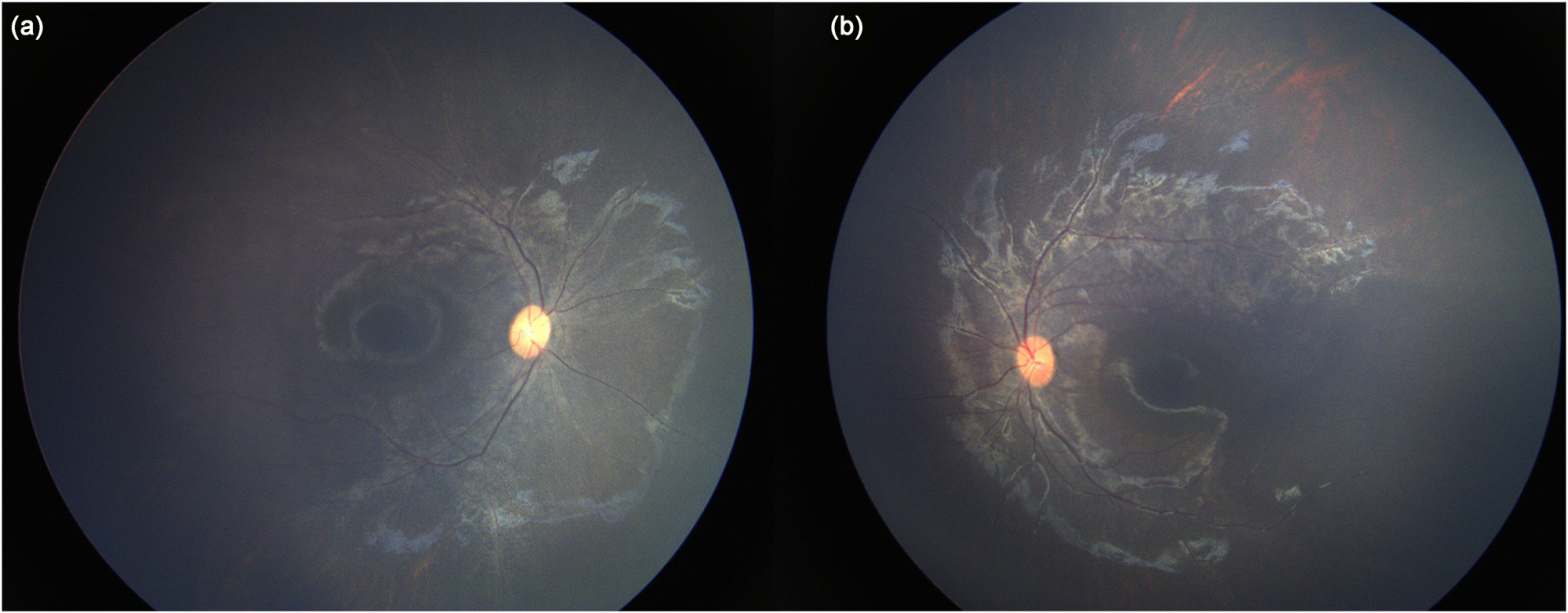
Fundus photography using Recam III system for a 2-year-old boy in the right eye (a) and left eye (b). Atrophic areas were originated from the optic papilla, which distributed in a radiated pattern along the venous vessels. The vascular surface was wrapped and covered by osteocytes-like pigmentation. The macular region was not involved.

A 2-year-old boy presented to our department due to photophobia for 17 months. There was no family history of ocular diseases (Figure 1). Physical examination was conducted to exclude the systemic diseases. Ophthalmic examination revealed +6.50DS/+1.50DC × 90° in the right eye and +6.50DS/+2.0DC × 90° in the left eye. The bilateral optic papilla was normal. Atrophic areas in a grey color were originated from the optic papilla, which distributed in a radiated pattern along the venous vessels. Besides, pigmentary deposition was seen along the venous vessels. The vascular surface was wrapped by osteocytes-like pigmentation, especially the venous region in the left upper eye. The macular region was not involved (Figure 2).

**Figure 3:**
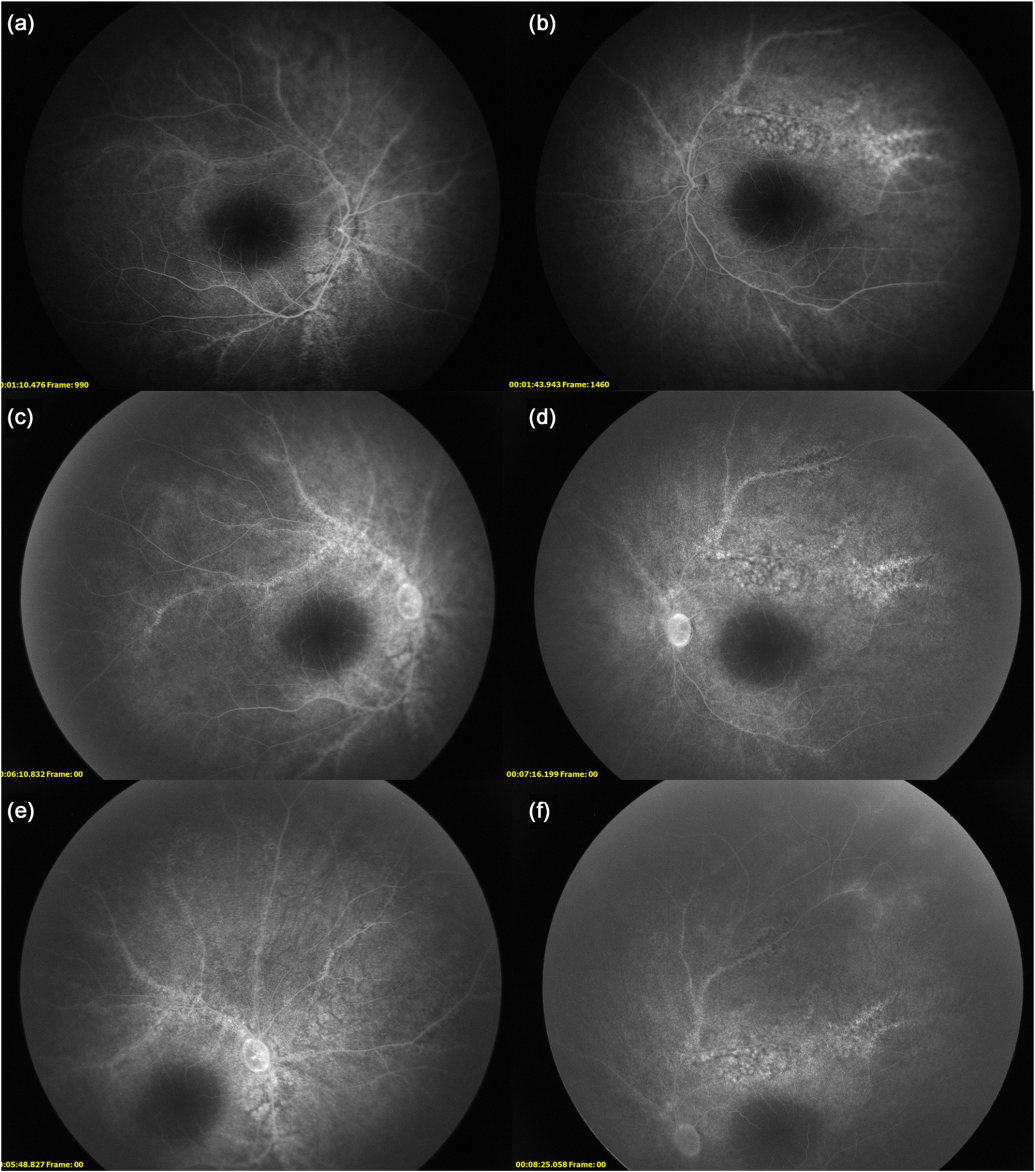
Findings of FFA. (a and b) FFA findings at venous phase in right eye and left eye. There was fluorescence in the atrophic area along the venous vessels originated from optic disk in both eyes. The fluorescence was covered by osteocytes-like pigmentation. The macular region was not involved. (c–f) Findings of FFA at advanced phase in right eye and left eye indicated no significant leakage in the posterior pole. Besides, staining and leakage were seen in the peripheral vessels in both eyes.

After obtaining the informed consent from the parents, fluorescein fundus angiography (FFA) was performed, which showed transmitted fluorescence in the atrophic area along the venous vessels originated from optic disk at venous phase. In addition, transmitted fluorescence and marginal staining were noticed in a plaque-like pattern along the venous vessels. The fluorescence was covered in the pigmentation area (Figure 3). Moreover, there was no obvious leakage in the fluorescence in the posterior pole of the eyes and peripheral tissues. Staining was noticed in the peripheral vessels. Finally, the patient was diagnosed with PPRCA.

**Figure 4:**
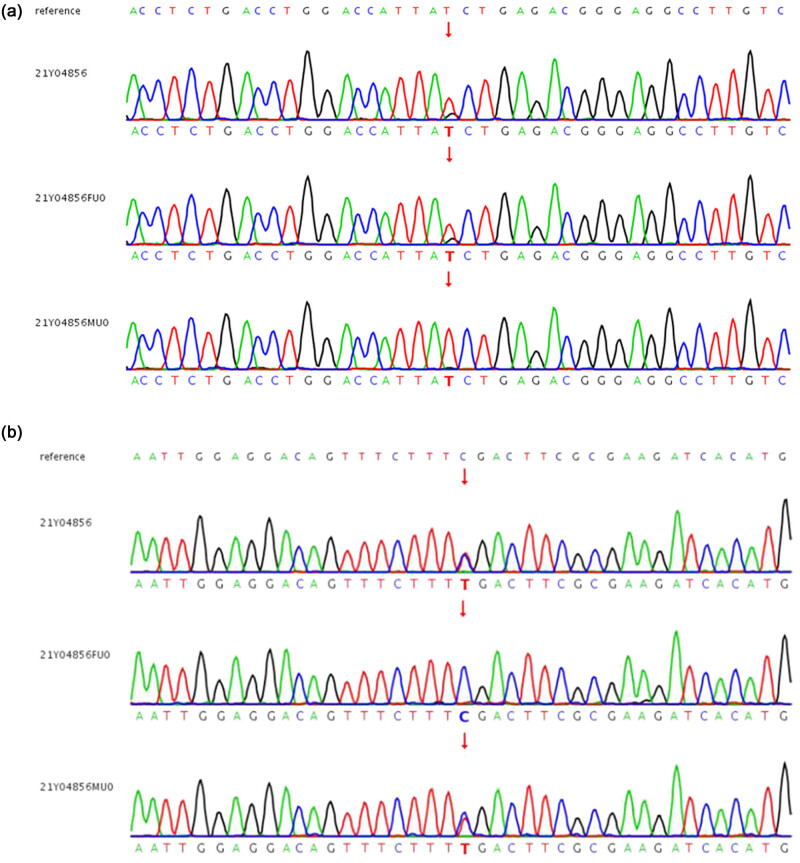
Sequence chromatograms of the family with c.2592T > G:p.Y864* mutation (a) and c.154C > T:p.R52* mutation (b) in *RPGRIP1*. 21Y04856: patient, 21Y04856FU0: father, and 21Y04856MU0: mother.


A Trio-WES strategy was utilized to identify the causal variants according to the previous description [6]. The Verita Trekker^®^ Variants Detection System (Berry Genomics, Beijing, China) was employed for the variant calling. The libraries were quantified by qPCR and size distribution was determined using Bioanalyzer 2100 device (Agilent Technologies, Santa Clara, CA, USA). Finally, Novaseq 6000 platform (Illumina, San Diego, USA), with 150 bp pair-end sequencing mode, was used for sequencing the genomic DNA of the family. Raw image files were processed using CASAVA v1.82 for base calling and generating raw data. WES identified two heterozygous truncation variants within NM_020366.4: c.2592T > G: p.Y864* and NM_020366.4: c.154C > T: p.R52* located in exon 17 and exon 3, respectively. Sanger confirmation of the variants obtained from WES was done through Berry Genomics with specific primers (Table A1, Figure 4a and b). Based on the ACMG criteria, the variants were considered to be pathogenic for the pathogenesis of PPRCA. The mode of inheritance was autosomal recessive.

**Informed consent:** Informed consent has been obtained from all individuals included in this study.
**Ethical approval:** The research related to human use has been complied with all the relevant national regulations, institutional policies and in accordance with the tenets of the Helsinki Declaration, and has been approved by the Medical Ethics Committee of Kunming Children’s Hospital (Approval No. 2021-03-298-K01).

## 3 Discussion

Sporadical occurrence is reported in the majority of PPRCA patients, while some studies proposed a congenital origin based on few cases reporting a family history. In a previous study, Bozkurt et al. reported a mildly affected and asymptomatic 54-year-old PPRCA mother, along with her mildly affected daughter and severely affected 28-year-old son (proband) [7]. On this basis, there has been speculation as to the inheritance by autosomal dominant, recessive, X- and Y-linked patterns of transmission. Unfortunately, there is still no convincing evidence for any of the transmission mode. In this study, we reported a PPRCA patient carrying two pathogenic variants in *RPGRIP1* gene based on WES, and the inheritance was classified into autosomal recessive.

*RPGRIP1* gene, also known as Leber Congenital Amaurosis 6 (LCA6) gene, encodes a photoreceptor protein composed of 1,287 amino acids, which can interact with retinitis pigmentosa GTPase regulator protein [8]. Some functional studies have focused on the roles of *RPGRIP1* gene in animals. In a previous study, Won et al. established a mice model of RPGRIP1 carrying a splice acceptor site mutation in Rpgrip1 (nmf247), which showed impairment in rod photoreceptor outer segment elaboration and morphogenesis [9]. In addition, the RPGRIP1-deficient dogs showed a severe cone–rod dystrophy similar to that seen in humans [10]. In a canine model with *RPGRIP1* mutation, the animals showed cone–rod dystrophy [11]. Nowadays, *RPGRIP1* gene mutations have been screened in several ocular diseases, such as retinitis pigmentosa [12], cone–rod dystrophy [13,14], LCA [15–18], primary open angle glaucoma [19], and congenital retinopathies [20]. For example, Gerber et al. [8] identified homozygosity for c.3341A > G mutation in exon 21 of the *RPGRIP1* gene, resulting in Asp1114Gly substitution in the RPGR-interacting domain in patients with LCA. In three Pakistani families with probands of cone–rod dystrophy, Hameed et al. reported a c.1639G > T mutation in exon 13 and c.2480G > T mutation in exon 16 of the *RPGRIP1* gene, which led to changes in the casein kinase II phosphorylation site and CK2 domain of the RPGRIP1 protein, respectively [14]. Moreover, *RPGRIP1* gene mutations including c.1767G > T, c.1793G > A, and c.1904C > g have been considered to be related to the pathogenesis of primary open angle glaucoma by causing physical impairment of the interaction of RPGRIP1 protein with the other proteins [19]. To date, 104 missenses/nonsense variants have been identified in the HMGD database, but little is known about their potential roles in the pathogenesis of PPRCA. In this study, WES screened two pathogenic variants within *RPGRIP1* in a Chinese pediatric patient, including NM_020366.4: c.2592T > G: p.Y864* and NM_020366.4: c.154C > T: p.R52* in exon 17 and exon 3, which resulted in termination codons, respectively. In a previous study, lack of exon 17 in *RPGRIP1* would cause a reading frame shift (i.e., p.D905SfsX6) that was expected to result in loss of more than one-third of the RPGRIP1 protein [21]. On this basis, these pathogenic variants would cause functional changes of the *RPGRIP1* gene, which then affect the pathogenesis of PPRCA accordingly to the ACMG criteria.

PPRCA should be differentially diagnosed with the gyrate atrophy of the choroid and retina, retinitis pigmentosa, serpiginous choroidopathy, choroideremia, cone red dystrophy (CRD), and retinitis punctata albescens (RPA), respectively [22,23]. To obtain a comprehensive understanding on the variations of *RPGRIP1* in the pathogenesis of the ocular diseases, we searched the PubMed, Medline, and Embase for the related articles with the following key words: “*RPGRIP1*,” “c.2592T > G: p.Y864*,” “c.154C > T: p.R52*.” Finally, these two truncation variants have been reported to be associated with autosomal recessive disorders namely CRD [17] showing c.2592T > G: p.Y864*, as well as RPA [24] and retinitis pigmentosa [24] showing c.154C > T: p.R52*, respectively. For the differential diagnosis, patients with RPA are more likely to present night blindness at an early age, with white spots dispersed in the retina after fundus examination. Usually, the macular region was not involved. Unlike the RPA, PPRCA patients showed lesions with retinal atrophy in the surrounding vessels, with no white spots. CRD is usually featured by epiretinal photoreceptor atrophy in the macular region, in which the lesions increased with the aging process. The peripheral tissues and the whole retina may be involved. The symptoms were featured by elimination of glisten in the central fovea of macula, together with deletion of the pigmentary epithelium. In contrast, patients with PPRCA seldom showed macular involvement, with most of the lesions being quiescent. In this study, the patient was finally diagnosed with PPRCA after differential diagnosis based on the absence of characteristic correlation between vascular distribution and osteocytes-like pigmentation. The patient was compound heterozygous for two nonsense variants in *RPGRIP1*, and thus the mode of inheritance was autosomal recessive. This would enhance our understanding on the roles of *RPGRIP1* in the pathogenesis of PPRCA.

Nowadays, WES has been utilized for the screening of variants in genes associated with ocular diseases. For example, Bryant et al. used WES on a cohort of 69 patients with various forms of retinal degeneration, which reported likely pathogenic variants in 64% of the subjects [25]. The largest WES study in age-related macular degeneration was performed by Corominas et al. in a large European cohort consisting of 1,125 age-related macular degeneration patients and 1,361 control participants, which screened a rare variant in *COL8A1* serving as a component of Bruch’s membrane [26]. However, few studies have utilized the WES for screening pathogenic variants in PPRCA. In this study, we reported a case with PPRCA, and WES was performed to screen the pathogenic variations accordingly. Two mutations resulting in terminal codon were screened. In the future, WES could be useful for genetic diagnosis and for identifying the defective gene region in patients with retinal degenerative diseases that are difficult to differentiate on the basis of clinical findings alone.

Indeed, there are some limitations for this study. First of all, the sample size is not large due to disease rarity. Second, although we screened two gene mutation yielding terminal codon in the *RPGRIP1* gene, we cannot find out the exact mechanism involved in this process.

## 4 Conclusion

We screened two pathogenic variants in the *RPGRIP1* gene in a 2-year-old boy with PPRCA based on WES including NM_020366.4: c.2592T > G: p.Y864* and NM_020366.4: c.154C > T: p.R52* in exon 17 and exon 3. The termination codon would significantly lead to protein structure changes, which then involve in the pathogenesis of PPRCA.

## Appendix

**Table A1:** PCR amplification conditions for the RPGRIP1

Variant	Exon	Primer sequence	Annealing temperature (°C)	Variation type
NM_020366.4: c.2592T > G:	17	Forward: 5′-TGCCATCATTCCAGCCAGT-3′; Reverse: 5′-TCTTCTGCTCTGTTGCTCTTGAC-3′	60	Pathogenic
NM_020366.4: c.154C > T	3	Forward: 5′-TGCTCTCTGGACAAGATGTGATGA-3′; Reverse: 5′-CTATCTCCATCCCCTCAGTTGTG-3′	60	Pathogenic
